# Lateral foot-use asymmetry and offensive effectiveness in elite 5-a-side soccer for the blind: an exploratory notational analysis

**DOI:** 10.3389/fspor.2026.1887871

**Published:** 2026-10-21

**Authors:** Boryi A. Becerra-Patiño, José Pino-Ortega, Rodrigo Yáñez-Sepúlveda, José Francisco López-Gil

**Affiliations:** 1 Programa de Doctorado en Ciencias de la Actividad Física y del Deporte, University of Murcia, San Javier, Murcia, Spain; 2 Faculty of Physical Education, National Pedagogical University, Bogotá, Colombia; 3 Faculty of Sport Science, University of Murcia, Murcia, Spain; 4 Faculty of Education and Humanities, School of Sport Sciences, Universidad Andrés Bello, Viña del Mar, Chile; 5 Department of Sport Sciences, Faculty of Sport and Health Sciences, Fit Generation Research Institute, Andorra la Vella, Andorra; 6 School of Medicine, Universidad Espíritu Santo, Samborondón, Ecuador; 7 Vicerrectoría de Investigación y Postgrado, Universidad de Los Lagos, Osorno, Chile

**Keywords:** adapted sport, blind football, foot preference, laterality, notational analysis, technical–tactical performance, paralympic sport

## Abstract

**Background:**

In 5-a-side soccer for the blind (F5), functional laterality takes on a particular significance distinct from that in conventional soccer because players do not visually choose which leg to use; rather, they orient themselves physically through auditory and tactile cues developed over years of training. However, how often each foot is used in competition, and whether the executing foot is associated with offensive effectiveness, have not been previously quantified.

**Objective:**

To quantify foot-use asymmetry at the team and individual levels in the Argentine men's national F5 team during the 2022 International Blind Sports Federation (IBSA) Copa América and to explore whether the executing foot was associated with offensive effectiveness beyond the spatial and contextual characteristics of each action.

**Methods:**

A retrospective notational analysis of a publicly available event log covering six matches was conducted. After removal of 14 duplicated goal records and eight actions without a recorded executing foot and one body-executed action, 141 shooting actions by eight players were analysed (139 with court coordinates). Foot-use asymmetry was quantified with an asymmetry index (AI). Goal and on-target rates were compared with Fisher's exact test, and associations with goal scoring were estimated with Firth-penalised logistic regression, with sensitivity analyses addressing clustering within players and individual foot preference.

**Results:**

The team showed marked right-foot predominance (107 right-footed versus 34 left-footed actions; AI = 51.8%), and five of the eight players used a single foot exclusively. Left-foot actions had a higher goal rate than right-foot actions: 17.6% compared with 7.5% [odds ratio (OR) = 2.65; 95% confidence interval (CI): 0.85–8.28; *p* = 0.102]. The estimate was similar after adjustment for origin of the play, lateral lane, distance from the goal and minute of play (Firth OR = 3.25; 95% CI: 0.96–11.35; *p* = 0.057), but it was imprecise and not statistically significant in any specification. Four of the six left-foot goals were scored by the only exclusively left-footed player; when actions were classified relative to each player's own preferred foot, the association was weaker (non-preferred versus preferred foot: 16.7% versus 9.3%; OR = 2.24; 95% CI: 0.40–8.96; *p* = 0.322). Shot distance and shot angle did not differ between feet and were not associated with goal scoring; 94.2% of shots were taken within 10 m of the goal line.

**Conclusions:**

The Argentine team combined marked team-level right-foot predominance with pronounced individual foot specialisation. The higher crude goal rate of left-foot actions was imprecise, was largely attributable to one left-footed player and was weaker when foot use was expressed relative to individual preference; it should therefore be regarded as a hypothesis to be confirmed in larger, multi-team samples rather than as evidence of a non-dominant-limb advantage. Individual foot-use profiles may nonetheless inform technical–tactical scouting and the planning of bilateral training in athletes with visual impairments.

## 1 Introduction

5-a-side soccer for the blind (F5) has been a Paralympic sport since the 2004 Athens games, and scientific literature has established that physical and technical-tactical variables are key determinants of performance (1). These physical and technical-tactical variables, which include postural control, balance, and the accuracy of shots and actions created in offensive zones, have made it possible to analyse performance in this sport (2–5).

Players with blindness show specific postural-control characteristics (6) and proprioceptive adaptations (7). In parallel, interlimb asymmetry, that is, the comparative analysis of the performance of one leg relative to the other, is a topic that has garnered significant attention in scientific literature (8, 9). Some studies have determined that asymmetries influence the athletic performance of players in team sports (10–12). In sports such as F5, in which game situations are constantly changing due to the sport's unpredictability, a strong reliance on one side may accentuate these imbalances (13). Several studies report that these imbalances may increase during athletic training due to the high volume of actions performed with the preferred side versus the non-preferred side (14, 15). Certain competitive actions involve unilateral and asymmetrical movements, all of which are associated with technical-tactical actions in offensive and defensive situations (16, 17).

Scientific literature on technical-tactical variables has revealed that the effectiveness of shots, as well as ball control and dribbling, are actions that influence player performance (18, 19). The analysis of these variables is determined primarily using video analysis, particularly the observational instrument for evaluating goal-scoring attempts in F5 for the blind (IOLF5C) (19, 20), a scientifically validated tool for evaluating effectiveness and key game characteristics as well as laboratory systems for motion capture and analysis via simulated vision (21–23).

Other studies have focused on analysing contextual variables by evaluating different moments of the match, playing time, and sporting result; game variables through analysis of the play initiation zone; characteristics of the progression; and variables related to shots on goal, including the evaluation of the leg used, the contact surface, and the level of opposition, most of which are in official matches (24, 25). Technical-tactical actions should be understood as the player's ability to know what to do at every moment of the game (26, 27), especially in a sport with high levels of uncertainty resulting from players’ lack of vision. In this context, some studies have established that the relationships between certain technical variables particularly shooting on goals and the tactical knowledge demonstrated by the player in competition are essential factors for understanding performance (28, 29) and, on the basis of this, for adapting specific teaching-learning processes to each context (30) and according to the demands of each sport, in this case, F5. However, despite growing interest in studying how technical-tactical variables influence athletes’ performance in team sports such as soccer and conventional futsal (31–33), little attention has been given to evaluating these variables in F5.

Notably, in F5, laterality takes on an additional dimension. Unlike conventional soccer or futsal, where the visual choice of which leg to use can be modified in fractions of a second depending on the game context to make decisions (34–36), F5 players rely solely on acoustic and proprioceptive information to orient their bodies relative to the audible ball. Consequently, the leg used to execute each action is a direct consequence of the body's orientation at the moment of contact with the sound-emitting ball, an orientation shaped by the auditory cues provided by the guides’ voices. This is how players develop motor patterns that are consolidated over years of training (37). Based on the above, the asymmetry observed in F5 may reflect not only tactical choices but also motor patterns consolidated through training and competition, which could, in principle, be modified by specific practice.

Similarly, there is a question that has been explored little in scientific literature regarding whether the less frequently used foot differs in effectiveness from the preferred foot when generating actions that result in goals or shots on goal. Consequently, to the best of our knowledge, no studies have been conducted to analyse offensive laterality in F5 players. Thus, the objective of the present study was to quantify foot-use asymmetry at the team and individual levels in the Argentine men's national F5 team during the 2022 International Blind Sports Federation (IBSA) Copa América and to explore whether the executing foot was associated with offensive effectiveness beyond the spatial and contextual characteristics of each action. We hypothesised that the team would exhibit a marked lateral asymmetry favouring the right foot and that, despite their lower frequency, left-foot actions would show a goal-conversion rate at least comparable to and potentially higher than that of right-foot actions, reflecting their possible use in less anticipated offensive situations. Because limb dominance was not assessed independently, foot use is described as right or left at the team level and relative to each player's observed preference (preferred or non-preferred foot) at the individual level.

## 2 Materials and methods

### 2.1 Design

A retrospective observational study (38) based on notational analysis was conducted on the six matches played by the Argentine men’s national F5 team at the 2022 IBSA Copa América, which took place between October 23 and 28, 2022 (Córdoba, Argentina).

### 2.2 Sample

The source event log contained 164 records for the Argentine team: 148 shooting actions from open play or restarts, 2 penalty kicks and 14 goal records. Each goal record duplicated an existing shooting or penalty record (identical match, minute, player, executing foot and outcome) and was therefore removed, leaving 150 unique shooting actions. Exclusion of eight actions without a recorded executing foot and one body-executed action yielded 141 foot-executed actions (107 right-footed and 34 left-footed) by eight players against Peru, Mexico, Chile, Colombia and Brazil (on two occasions: once in the group stage and once in the final). These actions produced 14 goals (8 right-footed, including 2 penalty kicks, and 6 left-footed); the team did not score against Brazil in either encounter, and the kicks of the penalty shoot-out that decided the final were not part of the event log. Because the 2 penalty kicks lacked court coordinates, all spatial and multivariable analyses were based on the 139 non-penalty actions with complete coordinates (12 goals). The championship was played according to IBSA regulations on a 40 × 20-meter court surrounded by kickboards, with four totally blind outfield players (IBSA B1 classification) wearing eyeshades plus a sighted goalkeeper per team.

### 2.3 Variables and coding

The variables were coded according to the following criteria:

- Executing foot (right, left or body)
- End-of-action court coordinates (X and Y) in meters on the regulation court (see below)
- Origin of the play (open play, high recovery, counterattack, set piece, penalty)
- Type of combination (individual or collective)
- Match minute, opponent, and player

Players were anonymised via sequential codes (P01–P08) in descending order based on the number of recorded actions.

The source database provides the end-of-action location of each shooting action as court coordinates in meters on the regulation 40 × 20 m court. The *X* axis corresponds to the lateral position across the court width (0–20 m, with the goal centre at X = 10 m), and the *Y* axis corresponds to the longitudinal position; for the attacks of the Argentine team, Y equals the distance from the attacked goal line (Y = 0 m) (Supplementary Material). The database also contains two further variables (Xi and Yi). These were almost perfectly collinear with X and Y (Pearson |r| > 0.9999; maximum residual 0.05 m, i.e., rounding), which shows that they are the pixel coordinates of the same end location on the digitisation template rather than the starting point of the play. Consequently, no start-of-play coordinates were available, and no progression-related variable could be derived. Because the left–right orientation of the digitisation template relative to the attacking players could not be verified from the source documentation, lateral position was analysed without assigning left or right wings (Section 2.4).

The origin-of-play categories were operationally defined as follows: open play, actions developed within continuous team possession with no preceding stoppage; set piece, actions originating from a restart of play (e.g., kick-ins, corner kicks and free kicks); counterattack, fast offensive transitions initiated immediately after regaining possession while the opponent was unbalanced; high recovery, actions initiated after regaining possession in the attacking half; and penalty, direct penalty kicks awarded under IBSA regulations. In the coding scheme, opponent denotes the rival national team faced in each match (Peru, Mexico, Chile, Colombia or Brazil), and player denotes the Argentine outfield player who executed the action, subsequently anonymised as described above.

The offensive actions analysed in this study derive from a publicly available, retrospective database (Supplementary material). The categorical variables in the database were the origin of the play, type of combination, foot used, and outcome of the shot; however, the actions and court coordinates were coded by the creator of the database rather than by the authors, and no information on the intra- or inter-observer reliability of the categorical coding or of the spatial digitisation is available. This is acknowledged as a limitation (Section 4.1).

### 2.4 Derived variables

The following variables were derived from the end-of-action coordinates: i) distance to the goal, computed as the distance from the shot location to the goal centre, √((X−10)^2^ + Y^2^); ii) absolute shot angle, i.e., the unsigned angle between the line joining the shot location to the goal centre and the longitudinal axis of the court (0° = frontal position; values increase towards the sidelines); and iii) court zones. Depth was divided into four bands (0–6 m, 6–10 m, 10–20 m, 20–40 m from the goal line), and lateral position into a central lane (7–13 m) and two wide lanes (0–7 m and 13–20 m), which were pooled for the inferential analyses because their left–right orientation could not be verified (Supplementary Material). Because these variables are deterministic geometric transformations of the court coordinates, they introduce no coding decisions beyond those already contained in the source database; their validity therefore depends on the accuracy of the digitised coordinates. They were incorporated to characterise the spatial context of each action, information not captured by the categorical codes of the database.

### 2.5 Asymmetry index

The lateral asymmetry index (AI), following the normalised interlimb AI approach used in sport laterality research (11), was calculated as AI = |n_R−n_L|/(n_R + n_L) × 100, where n_R and n_L correspond to the number of actions performed with the right and left legs, respectively. The AI can range from 0 (perfect symmetrical use) to 100 (exclusive use of one leg). It was calculated at the team level and for each player; correlational analyses were restricted to players with at least five recorded actions.

Because limb dominance was not assessed independently (e.g., by questionnaire or standardised kicking tests), each player's preferred foot was operationally defined as the foot used in the majority of his recorded actions, and every action was classified as executed with the preferred or the non-preferred foot. At the team level, the executing foot is therefore described as right or left, without assuming that the right foot is the dominant limb of every player.

### 2.6 Outcome variables

Two binary outcomes were defined. The primary outcome was a goal. The secondary outcome was a shot on target, defined as a goal or a shot saved by the goalkeeper; shots that were blocked, off target, hit the woodwork or were unsuccessful attempts (coded as “Fallo” in the source database) were coded as not on target.

### 2.7 Statistical analysis

Differences in goal and on-target rates between feet were tested via Fisher's exact test, which was preferable to the chi-square test because of the low expected frequency in some cells; crude odds ratios (ORs) are reported with Woolf (logit) 95% confidence intervals (CIs), and absolute differences in rates with Newcombe hybrid-score 95% CIs. Associations with goal scoring were modelled via logistic regression on the 139 non-penalty actions with complete coordinates, of which 12 resulted in a goal. The full model included the executing foot (left vs. right), the origin of the play (counterattack, high recovery and set piece vs. open play), the lateral lane (central vs. wide), the standardised distance to goal and the standardised match minute [seven parameters; ≈1.7 events per parameter, well below the 10 events per variable recommended by Peduzzi et al. (39)]. All logistic models were therefore estimated via Firth's penalised likelihood (40), which corrects the small-sample bias of maximum-likelihood estimates and remains stable under sparse data or quasi-separation; ORs are reported with profile penalised-likelihood 95% CIs, and significance was assessed via penalised likelihood-ratio tests. A reduced model including only the executing foot and the standardised distance to goal (≈6 events per parameter) was specified as the most stable adjusted estimate. The fit of the unpenalised full model is described via the Akaike information criterion (AIC) and the Cox–Snell and Nagelkerke pseudo-R^2^. Four sensitivity analyses addressed the non-independence of actions and the definition of the executing foot: (i) random-intercept logistic models with player or match as the clustering unit, estimated by maximum likelihood with 40-point Gauss–Hermite quadrature; (ii) leave-one-player-out re-estimation of the reduced Firth model; (iii) exclusion of the only exclusively left-footed player; and (iv) replacement of left/right foot with non-preferred/preferred foot. The association between the individual AI and the goal rate was examined via Pearson and Spearman correlations in players with at least five actions. Shot angle and distance were compared (in terms of stochastic superiority) between feet via the Mann‒Whitney U test, and the proportion of central-lane actions via Fisher's exact test. Given the exploratory nature of the study and the small number of goal events, emphasis is placed on effect estimates and their CIs rather than on statistical significance; *p* values are reported exactly, and *α* = 0.05 is used only as a descriptive reference. All analyses were performed in Python 3.12 (NumPy 2.4, pandas 3.0, SciPy 1.17, statsmodels 0.15) (41), with custom implementations of the Firth and random-intercept models; the complete, reproducible analysis code is provided as Supplementary Material.

### 2.8 Ethical considerations

This study used a publicly available event log derived from official IBSA Copa América broadcasts, which are in the public domain. Individual identities were replaced with sequential codes prior to analysis. The study followed the guidelines established by the Declaration of Helsinki (42) to ensure privacy and proper data handling, particularly for populations with disabilities.

## 3 Results

The following analyses are reported: i) team-level foot-use asymmetry; ii) effectiveness by executing foot; iii) individual-level asymmetry and foot preference; iv) multivariable and sensitivity analyses of goal scoring; v) spatial effectiveness stratified by foot; and vi) spatial characteristics of shots by foot.

### 3.1 Lateral asymmetry at the team level

Of the 141 analysed actions, 107 (75.9%) were executed with the right foot and 34 (24.1%) with the left foot, corresponding to an AI of 51.8% and a right-to-left ratio of 3.1:1 (Figure 1a). Figure 1b shows the goal and on-target rates by executing foot; the brackets report Fisher's exact tests for both outcomes.

**Figure 1:**
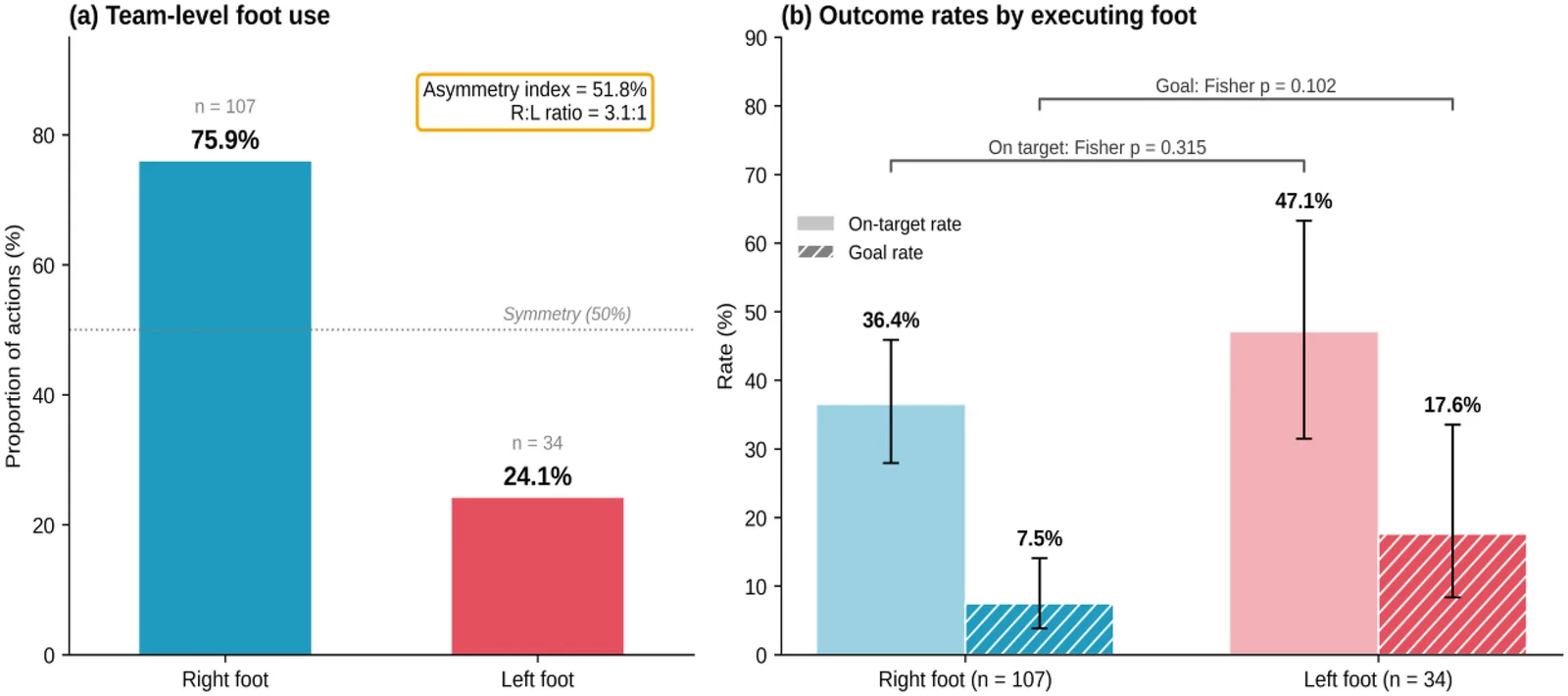
Foot-use asymmetry and outcome rates at the team level. **(a)** Distribution of the 141 foot-executed actions (107 right, 34 left). **(b)** Goal and on-target rates by executing foot with 95% Wilson confidence intervals; brackets report Fisher's exact tests. Fourteen duplicated goal records, eight actions without a recorded foot and one action executed with the body were excluded.

Table 1 shows the number of actions (n), goals and shots on target, the goal and on-target percentages, and the absolute shot angle and distance to goal of the non-penalty actions, stratified by the foot used to execute the action.

**Table 1:** Outcomes and spatial characteristics of actions by executing foot.

Foot	n	Goals	On-target	Goal rate (%)	On-target rate (%)	Angle, median (°)	IQR (°)	Distance, median (m)	IQR (m)
Right	107	8	39	7.5	36.4	34.1	20.6–51.5	7.2	5.8–9.0
Left	34	6	16	17.6	47.1	33.2	17.7–48.8	6.5	5.3–8.0

Angle, absolute shot angle relative to the goal axis; Distance, distance to the goal centre. Spatial variables were computed for the 139 non-penalty actions with court coordinates (105 right-footed, 34 left-footed). n, number; %, percentage; IQR, interquartile range; m, meters. Distance and angle compared between feet with Mann‒Whitney U test on medians (see Section 3.6).

### 3.2 Effectiveness by executing foot

Actions performed with the left foot showed a higher goal rate than those performed with the right foot, although the difference did not reach statistical significance: 17.6% (6/34) versus 7.5% (8/107), corresponding to an absolute difference of 10.1 percentage points (95% CI: −1.2 to 26.4) and a crude OR of 2.65 (95% CI: 0.85–8.28; Fisher's exact test, *p* = 0.102). The on-target rate showed the same direction: 47.1% with the left foot versus 36.4% with the right foot (OR = 1.55; 95% CI: 0.71–3.38; *p* = 0.315) (Figure 1b). In both cases the CIs were wide and compatible with no difference as well as with a substantial left-foot advantage. Spatially, the actions of both feet were concentrated close to the goal: 94.2% of the non-penalty actions were taken within 10 m of the goal line, and all goals were scored within this distance (Figure 2). Right-foot actions covered a wider range of lateral positions than left-foot actions (standard deviation [SD] of lateral position, 4.5 versus 3.3 m), which is consistent with their higher frequency of use.

**Figure 2:**
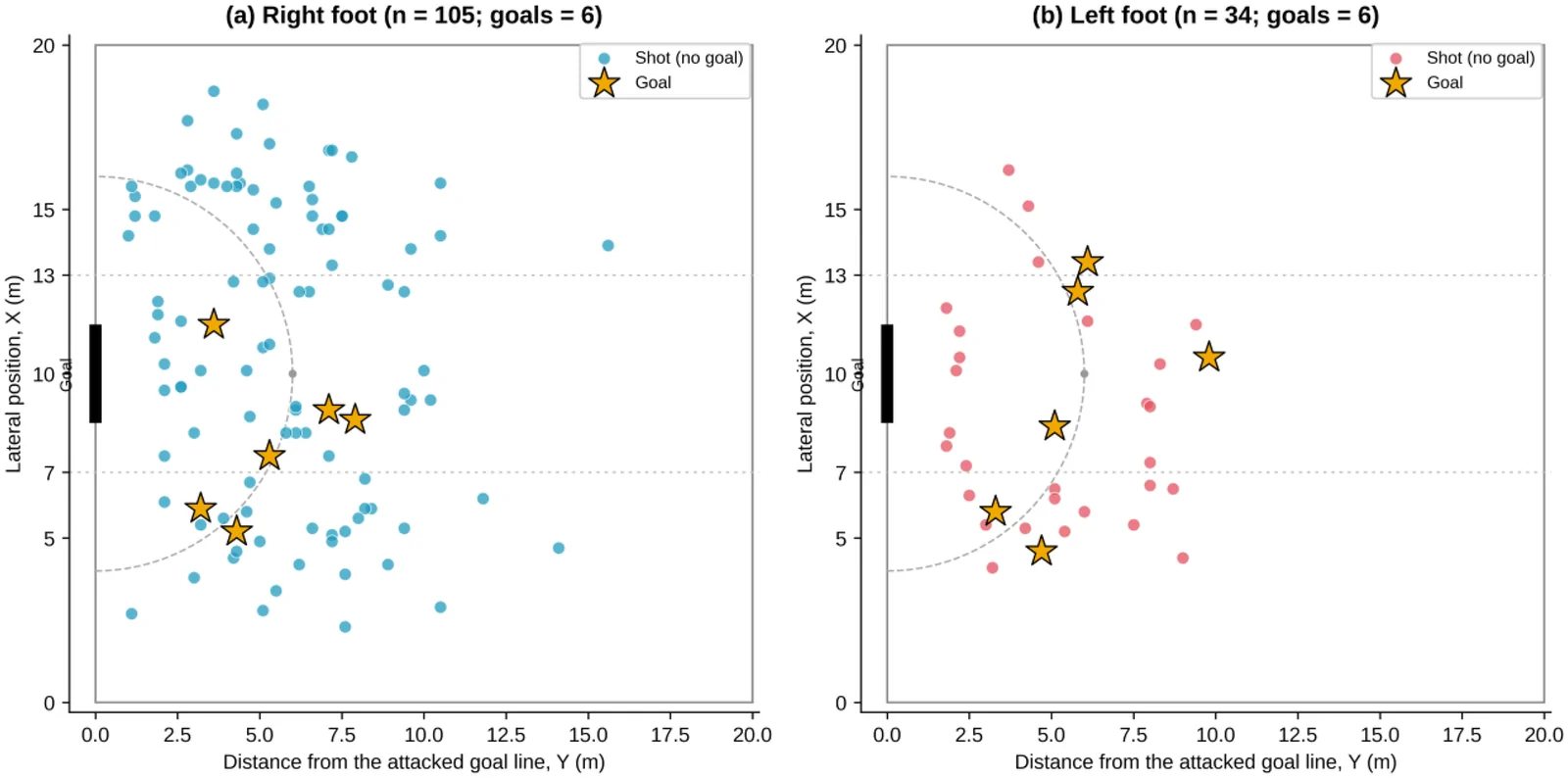
Spatial distribution of non-penalty actions by executing foot. Panels show **(a)** right-foot actions (*n* = 105; 6 goals) and **(b)** left-foot actions (*n* = 34; 6 goals); stars mark goals. The goal is at the left edge (Y = 0 m); the dashed arc marks 6 m from the goal centre, and the dotted lines delimit the central lane (X = 7–13 m). The two right-footed penalty goals are not shown because penalty kicks lacked court coordinates.

### 3.3 Individual-level asymmetry and foot preference

Interindividual heterogeneity was marked (Figure 3a). Five of the eight players (62.5%) had AI = 100%, meaning that they used a single foot throughout the tournament: four used only the right foot (P04, P05, P07, P08) and one only the left foot (P03; *n* = 22 actions, with a goal rate of 18.2%). Another player was almost exclusively right-footed (P02 with AI = 91.7%). Only two players (P01 and P06) used both feet regularly, with AIs of 54.3% and 40.0%, respectively. The association between the individual AI and the goal rate was not statistically significant (Figure 3b; Pearson r = 0.30; *p* = 0.520; Spearman *ρ* = 0.29; *p* = 0.531; k = 7). The players with the most bilateral use (P01, P06) presented goal rates of 2.9% and 10.0%, whereas players who used a single foot presented rates ranging from 0% (P07, P08) to 20.0% (P04). With only seven players, these data can neither support nor exclude a relationship between lateral specialisation and offensive performance.

**Figure 3:**
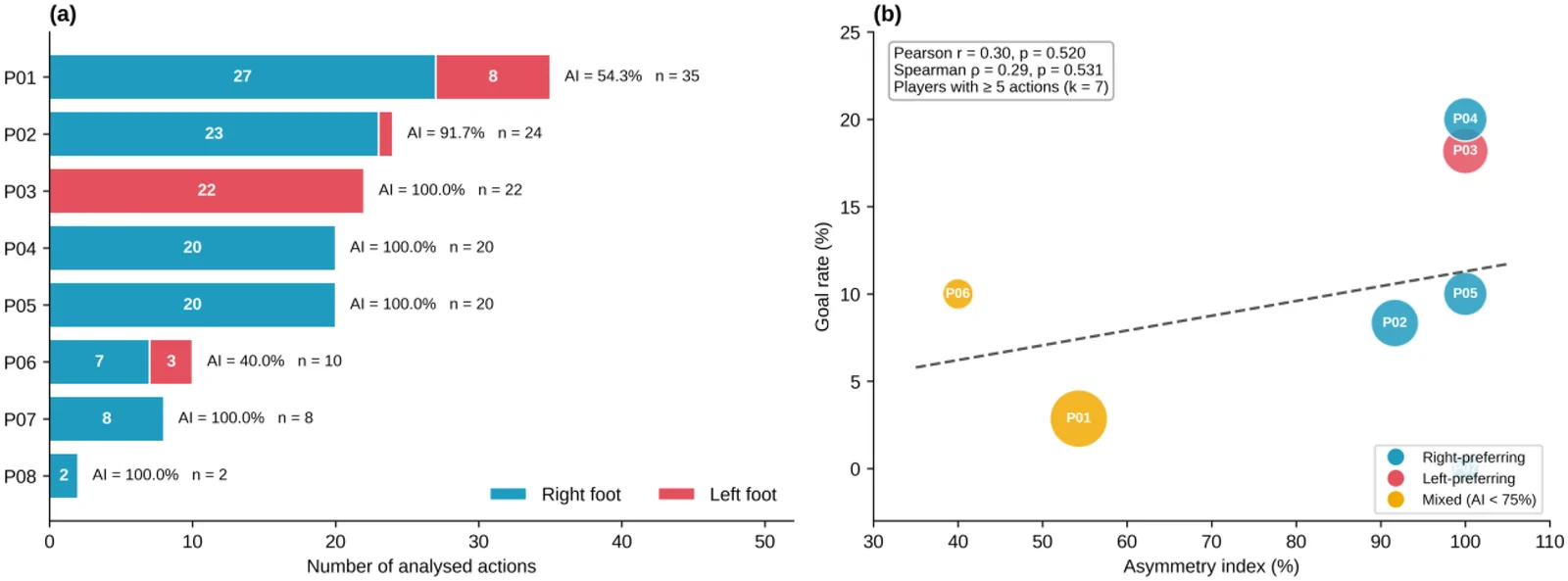
Interindividual variability in foot-use asymmetry. **(a)** Per-player foot-use composition with the corresponding asymmetry indices (AI) and sample sizes. **(b)** Scatterplot of the AI versus goal rate for players with at least 5 actions (k = 7); the bubble area encodes the sample size, and the dashed line shows the least-squares fit. Colours indicate the observed foot preference (right-preferring, left-preferring, or mixed when AI < 75%).

Table 2 provides a breakdown of the key variables for each of the 8 players analysed based on their foot usage distributions and success rates. The players are listed anonymously and ranked by the total number of recorded actions.

**Table 2:** Player-level laterality and outcome rates.

Player	*n*	Right (n)	Left (n)	Asymmetry index (%)	Goal rate (%)	On-target rate (%)
P01	35	27	8	54.3	2.9	37.1
P02	24	23	1	91.7	8.3	41.7
P03	22	0	22	100.0	18.2	36.4
P04	20	20	0	100.0	20.0	45.0
P05	20	20	0	100.0	10.0	45.0
P06	10	7	3	40.0	10.0	50.0
P07	8	8	0	100.0	0.0	12.5
P08	2	2	0	100.0	0.0	0.0

n, number; %, percentage.

Because the left foot was the preferred foot of P03, left-foot actions were not equivalent to non-preferred-foot actions. Of the six left-foot goals, four were scored by P03 with his preferred foot and two by right-preferring players (P01 and P06) with their non-preferred foot. When each action was classified relative to the player's own preferred foot, non-preferred-foot actions (*n* = 12, all left-footed, performed by P01, P02 and P06) showed a goal rate of 16.7% (2/12) versus 9.3% (12/129) for preferred-foot actions (Firth OR = 2.24; 95% CI: 0.40–8.96; *p* = 0.322) and an on-target rate of 66.7% versus 36.4% (*p* = 0.061). Within the three players who used both feet, the non-preferred foot produced 2 goals from 12 actions, whereas the preferred foot produced 2 goals from 57 actions, both from penalty kicks (0 of 55 in open play).

### 3.4 Multivariable and sensitivity analyses of goal scoring

In the full Firth-penalised model (*n* = 139 non-penalty actions; 12 goals; Table 3; Figure 4a), the estimate for the left foot (vs. right foot) was similar in magnitude to the crude association (OR = 3.25; 95% CI: 0.96–11.35; *p* = 0.057). None of the covariates was associated with goal scoring: distance to goal [OR = 1.03 per +1 SD (2.6 m); 95% CI: 0.52–1.97; *p* = 0.938], central versus wide lane (OR = 2.16; 95% CI: 0.59–8.41; *p* = 0.242), origin of the play (all *p* ≥ 0.566) and match minute (OR = 0.87 per +1 SD; 95% CI: 0.46–1.57; *p* = 0.642). The unpenalised version of this model showed a modest fit (AIC = 91.4; Cox–Snell pseudo-R^2^ = 0.045; Nagelkerke pseudo-R^2^ = 0.101).

**Table 3:** Full Firth-penalised logistic regression model for goal scoring.

Predictor	*β*	SE	Odds ratio	95% CI	*p* value
Intercept	−2.959	0.641	0.05	0.01–0.16	< 0.001
Left foot (vs. right)	1.180	0.605	3.25	0.96–11.35	0.057
Origin: counterattack (vs. open play)	0.493	0.843	1.64	0.26–8.11	0.566
Origin: set piece (vs. open play)	0.030	0.731	1.03	0.22–4.41	0.968
Origin: high recovery (vs. open play)	−0.034	0.848	0.97	0.15–4.75	0.968
Lateral lane: central (vs. wide)	0.771	0.646	2.16	0.59–8.41	0.242
Distance to goal (per +1 SD)	0.026	0.324	1.03	0.52–1.97	0.938
Match minute (per +1 SD)	−0.143	0.297	0.87	0.46–1.57	0.642

SE, standard error (penalised information matrix); CI, confidence interval (profile penalised likelihood); *p* values from penalised likelihood-ratio tests; SD, standard deviation (distance to goal, 2.6 m; match minute, 9.3 min); β, log-odds coefficient; vs., versus.

**Figure 4:**
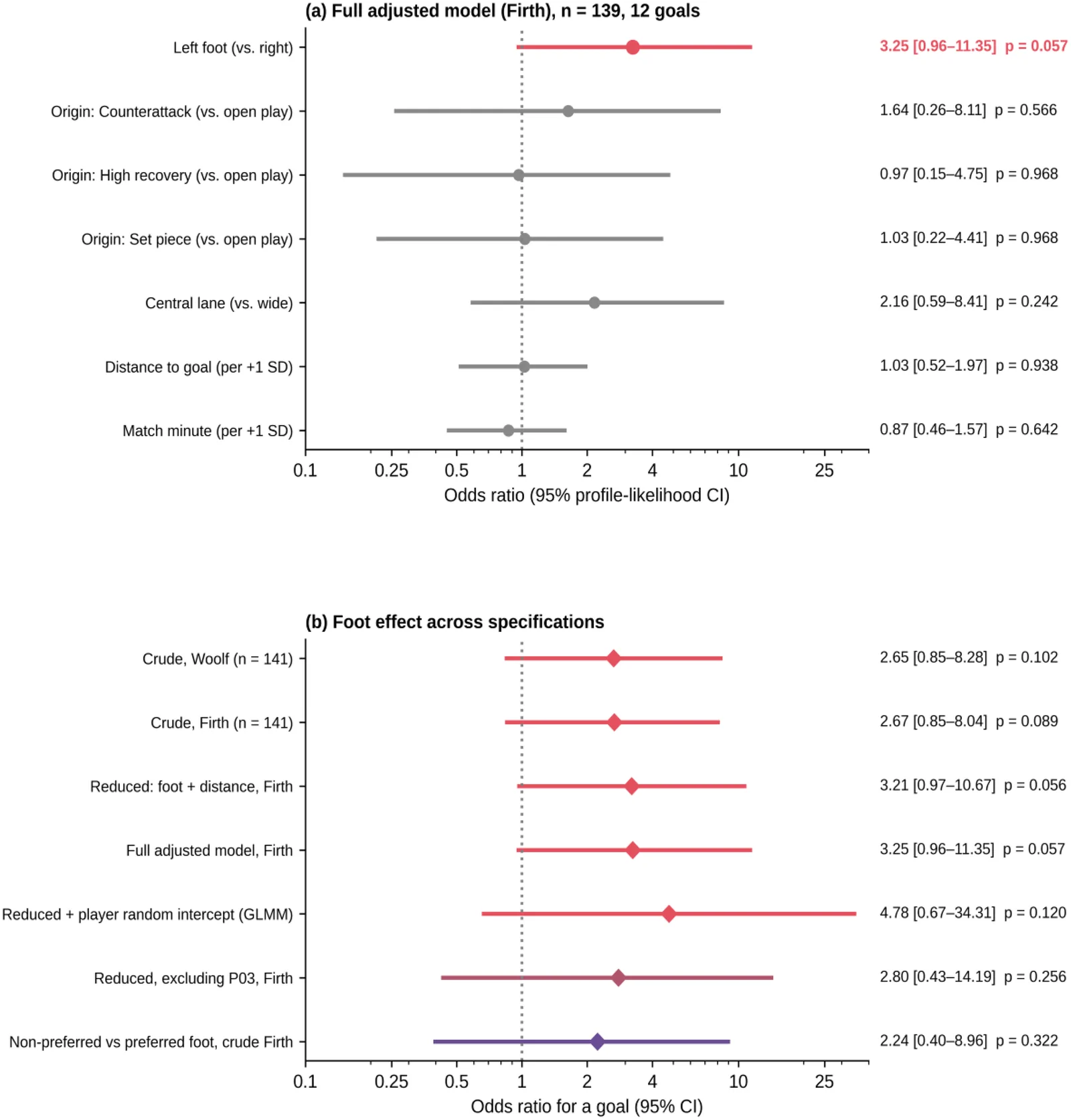
Odds ratios for goal scoring. **(a)** Full Firth-penalised logistic regression model (*n* = 139 non-penalty actions; 12 goals) with profile penalised-likelihood 95% CIs; reference categories: right foot, open play and wide lane; continuous predictors standardised (per +1 SD). **(b)** Estimates of the foot effect across analytical specifications: crude (Woolf CI and Fisher's exact test; Firth with profile-likelihood CI), reduced and full Firth models, random-intercept model with player as the clustering unit (Wald CI), reduced model excluding the exclusively left-footed player (P03), and non-preferred versus preferred foot (crude, Firth). GLMM, generalised linear mixed model.

To aid interpretation, at the reference covariate profile (open play, wide lane, mean distance and mean match minute), the model-predicted goal probability was 4.9% for a right-footed action and 14.4% for an otherwise identical left-footed action, an absolute difference of approximately 10 percentage points that mirrors the unadjusted difference (7.5% versus 17.6%). With ≈1.7 events per parameter, however, the adjusted estimates describe only the direction and approximate magnitude of the association.

The foot estimate was consistent in direction but imprecise across all specifications (Figure 4b; Table 4). The reduced Firth model (executing foot and distance to goal; ≈6 events per parameter) yielded OR = 3.21 for the left foot (95% CI: 0.97–10.67; *p* = 0.056), with no association for distance to goal (OR = 0.84 per +1 SD; 95% CI: 0.44–1.56; *p* = 0.579). Allowing for the clustering of actions within players (random-intercept model; between-player SD = 0.82 on the logit scale; intraclass correlation = 0.17) widened the interval considerably (OR = 4.78; 95% CI: 0.67–34.31; *p* = 0.120), whereas the match-level variance was estimated as zero, so that the match-clustered model reproduced the unpenalised reduced model. Leave-one-player-out estimates ranged from 2.49 (excluding P02) to 7.22 (excluding P04) and reached *p* < 0.05 only when a single right-footed scorer (P04 or P05) was removed; excluding the exclusively left-footed P03 reduced the estimate to OR = 2.80 (95% CI: 0.43–14.19; *p* = 0.256). Finally, when the executing foot was expressed relative to individual preference, the association was weaker (non-preferred vs. preferred foot, OR = 2.24; 95% CI: 0.40–8.96; *p* = 0.322). Taken together, no specification provided statistically significant evidence of a foot effect, and the point estimate depended strongly on the contribution of individual players.

**Table 4:** Sensitivity analyses of the association between executing foot and goal scoring.

Specification	n (goals)	Odds ratio	95% CI	*p* value
Crude, left vs. right (Woolf CI; Fisher's exact test)	141 (14)	2.65	0.85–8.28	0.102
Crude, left vs. right (Firth)	141 (14)	2.67	0.85–8.04	0.089
Reduced model: foot + distance (Firth)	139 (12)	3.21	0.97–10.67	0.056
Full model (Firth; Table 3)	139 (12)	3.25	0.96–11.35	0.057
Reduced model + player random intercept	139 (12)	4.78	0.67–34.31	0.120
Reduced model + match random intercept	139 (12)	3.30	0.97–11.27	0.056
Reduced model, leave-one-player-out (range)	104–137	2.49–7.22	—	0.007–0.256
Reduced model excluding P03 (Firth)	117 (8)	2.80	0.43–14.19	0.256
Non-preferred vs. preferred foot, crude (Firth)	141 (14)	2.24	0.40–8.96	0.322
Non-preferred vs. preferred foot + distance (Firth)	139 (12)	2.20	0.37–9.85	0.354

Odds ratios for a goal (left vs. right foot, or non-preferred vs. preferred foot). CI, confidence interval (profile penalised likelihood for Firth models; Wald for random-intercept models; Woolf for the crude odds ratio). The match-level variance was estimated as zero; hence, the match random-intercept model coincides with the unpenalised reduced model. P03: the only exclusively left-footed player.

Table 3 presents the coefficients, standard errors, odds ratios with profile penalised-likelihood 95% confidence intervals, and penalised likelihood-ratio *p* values of the full Firth-penalised logistic regression model with goal as the outcome variable (*n* = 139 non-penalty actions; 12 goals). Reference levels: right foot (executing foot), open play (origin) and wide lane (lateral position).

### 3.5 Spatial effectiveness stratified by foot

Conversion rates by zone are shown in Figure 5 and Table 5. Goals were concentrated close to the goal: the conversion rate was 10.1% (8/79) within 6 m of the goal line, 7.7% (4/52) between 6 and 10 m and 0% (0/8) beyond 10 m. For the right foot, conversion was 9.5% (2/21) and 14.3% (2/14) in the central lane at 0–6 m and 6–10 m, respectively, compared with 5.4% (2/37) and 0% (0/25) in the wide lanes at the same depths. Left-foot actions showed conversion rates between 14.3% and 22.2% in all four zones within 10 m; however, each of these zones contained only 6 to 12 actions and one or two goals, so zone-level estimates are highly unstable. The proportion of actions taken from the central lane did not differ significantly between feet (47.1% left versus 35.2% right; Fisher's exact test, *p* = 0.230).

**Figure 5:**
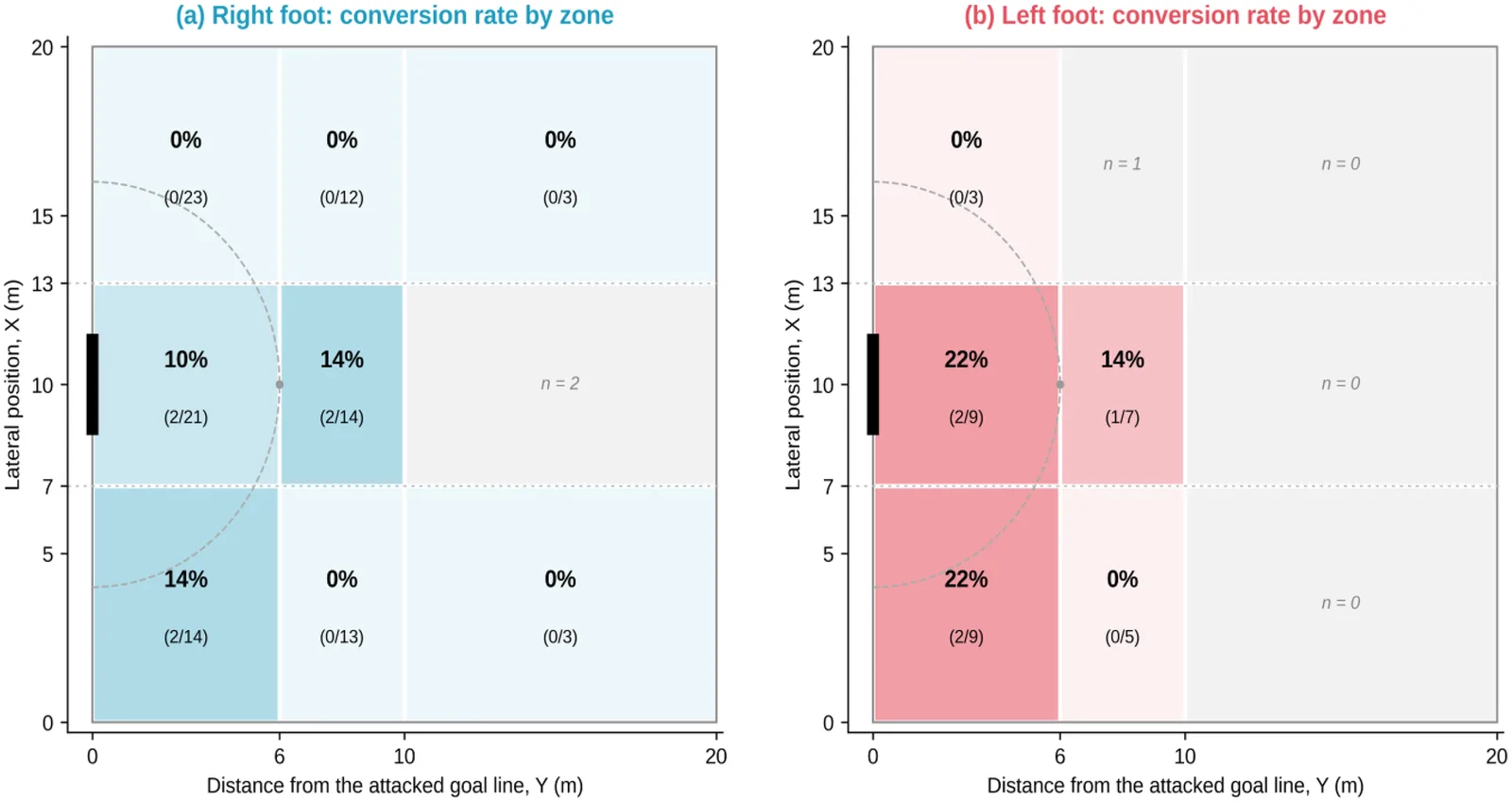
Foot-specific spatial efficacy. Conversion rate (goals/actions) by court zone for **(a)** right-foot and **(b)** left-foot non-penalty actions. Depth zones: 0–6, 6–10 and 10–20 m from the goal line; lateral lanes: X = 0–7, 7–13 and 13–20 m. Cells with fewer than three actions show the sample size only. Because the left–right orientation of the lateral lanes relative to the attacking players could not be verified, the two wide lanes are not labelled as left or right wings.

**Table 5:** Foot-specific zone-level efficacy.

Foot	Depth zone	Lateral lane	*n*	Goals	Conversion (%)
Right	Close (0–6 m)	Central	21	2	9.5
Right	Close (0–6 m)	Wide	37	2	5.4
Right	Mid-close (6–10 m)	Central	14	2	14.3
Right	Mid-close (6–10 m)	Wide	25	0	0.0
Right	Mid (10–20 m)	Wide	6	0	0.0
Left	Close (0–6 m)	Central	9	2	22.2
Left	Close (0–6 m)	Wide	12	2	16.7
Left	Mid-close (6–10 m)	Central	7	1	14.3
Left	Mid-close (6–10 m)	Wide	6	1	16.7

Central lane, X = 7–13 m; wide lanes, X = 0–7 m and 13–20 m (pooled). n, number; m, meters; %, percentage.

Table 5 presents the number of actions, goals and conversion rate for each foot   ×   depth zone   ×   lateral lane (central vs. wide) combination with at least three actions; combinations with fewer than three actions (*n* < 3) have been omitted.

### 3.6 Spatial characteristics of shots by foot

There was no evidence of stochastic superiority in shot angle between feet (median absolute angle, 33.2° [IQR 17.7–48.8] for the left foot versus 34.1° [IQR 20.6–51.5] for the right foot; *p* = 0.450) (Figure 6a). Left-foot actions tended to be taken slightly closer to the goal median 6.5 m [IQR 5.3–8.0] versus 7.2 m [IQR 5.8–9.0], although the difference was not statistically significant (*p* = 0.060) (Figure 6b).

**Figure 6:**
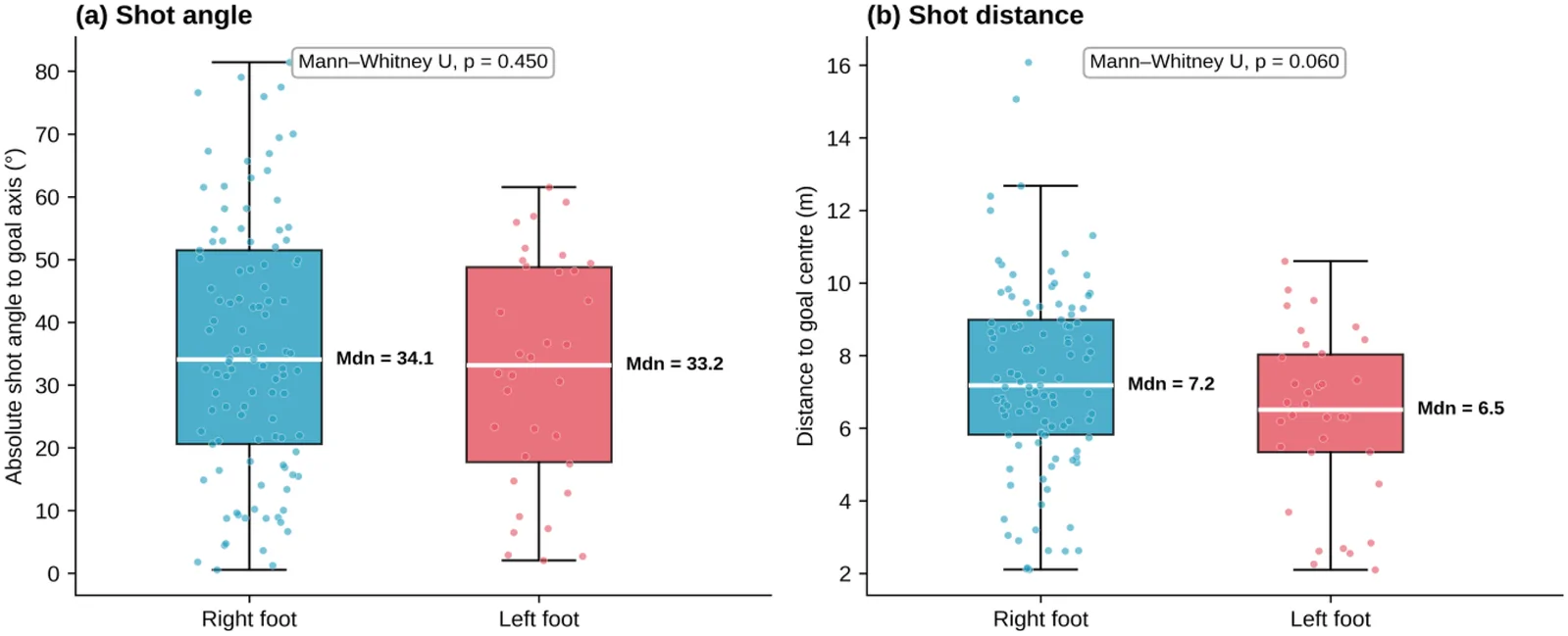
Spatial characteristics of non-penalty actions by executing foot. **(a)** Absolute shot angle relative to the goal axis (0° = frontal position). **(b)** Distance to the goal centre. Boxes show medians and interquartile ranges, whiskers extend to 1.5 × IQR, and dots represent individual actions; comparisons were performed via the Mann–Whitney U test.

## 4 Discussion

To the best of our knowledge, this is the first study to quantify foot-use asymmetry and its association with offensive effectiveness in an elite men's F5 national team. The data pertain to the Argentine national team, which won the 2022 IBSA Copa América, its fourth continental title. The main findings were a marked team-level right-foot predominance combined with pronounced individual specialisation, and a higher crude goal rate for left-foot actions that was imprecise, not statistically significant in any specification and largely attributable to a single left-footed player.

The asymmetry observed in the team (AI = 51.8%, R:L ratio of 3.1:1) falls within the range reported in soccer and futsal (15, 43, 44). Nevertheless, it should be interpreted differently from that in other team sports, such as soccer or futsal, since players with visual impairments (B1) do not have the ability to visually modify their decisions; rather, the executing foot is determined by the body orientation achieved within the acoustic-proprioceptive field. This is consistent with some studies that have sought to determine how asymmetries influence technical actions, finding that dribbling and balance are influenced by functional performance related to neuromuscular control and players’ motor skills (45).

Left-foot actions showed a higher crude goal rate than right-foot actions (17.6% vs. 7.5%; OR = 2.65; 95% CI: 0.85–8.28; *p* = 0.102), and the adjusted estimates were of similar magnitude (OR = 3.21–3.25). However, three features of the data argue against interpreting this as a non-dominant-limb advantage. First, the CIs were wide, compatible both with no effect and with a large effect, and no specification reached statistical significance. Second, the estimate depended strongly on individual players: four of the six left-foot goals were scored by the only exclusively left-footed player, for whom the left foot is the preferred foot, and leave-one-player-out estimates varied almost threefold. Third, when foot use was expressed relative to individual preference, the association was weaker and very imprecise (OR = 2.24; 95% CI: 0.40–8.96). The team-level left–right contrast therefore largely reflects squad composition, that is, which players are left- or right-footed and how effective they are, rather than a property of the limb used. This distinction matters when comparing our data with those of a study that analysed the FIFA Futsal World Cup®, which revealed that sighted players use their dominant limb more frequently for controlling and passing (*p* < 0.01; effect size (ES) > 0.39) and shoot with greater accuracy (*p* < 0.01; ES > 0.39), whereas shooting actions with the nondominant limb occurred at markedly lower frequencies (*p* < 0.01; ES > 0.62) (43). The small number of non-preferred-foot actions in our data (*n* = 12), two of which resulted in goals, is compatible with the hypothesis that F5 players use the non-preferred foot selectively, in situations in which body orientation relative to the acoustic field, the kickboards and the goal is favourable, or in which the sighted goalkeeper does not expect a shot from that side. However, these considerations are hypotheses rather than findings, as body orientation, defensive pressure and goalkeeper positioning were not measured. Some studies have analysed the prediction of actions based on position on the field, concluding that experience and the specific nature of the position on the field are essential factors in determining such predictions (46). Meanwhile, other studies have indicated that the goalkeeper's stance, lateral movement, and positioning are key factors in successfully stopping shots (47); whether similar mechanisms operate in F5, where only the goalkeeper is sighted, deserves specific investigation.

Spatially, the actions of both feet were concentrated close to the goal: 94.2% of the non-penalty actions were taken within 10 m of the goal line, all goals were scored within this distance, and shot distance and angle did not differ materially between feet. In contrast with the literature on sighted players, distance to goal was not associated with goal scoring in this sample, which is probably explained by the very narrow range of shooting distances (interquartile range of approximately 3 m), which limits the ability to detect a distance gradient, together with the small number of goals. Similarly, the study conducted by Palucci Vieira et al. (43) revealed no significant differences in the distance from the goal for defining accurate and inaccurate actions with the dominant and nondominant limbs, whereas other studies of sighted futsal players found the most effective actions to be those performed with the dominant limb (43, 48), and studies analysing the Futsal World Cup have noted that the effectiveness of shots can also be linked to the game's outcome, although no significant differences were found in the areas where the shots were taken (49).

According to Lapresa et al. (50) approximately 90% of shots on goal in futsal are taken within the attack zone, indicating that the location in the field is what determines different rates of accuracy and effectiveness, although other studies have concluded that the use of the nondominant limb is associated with the tactical strategies presented by the game (51, 52). In the present study, right-foot actions covered a wider range of lateral positions than left-foot actions, and the proportion of central-lane actions was similar for both feet. This suggests that, in F5, the choice of the executing foot may depend less on identifying a wide variety of options than on how body orientation relates to the dynamics of the game (acoustic geometry of the ball, kickboards, proximity to the opponent and the goal), possibly combined with seeking the element of surprise to overcome an opposing defence that also moves on the basis of acoustic information and a goalkeeper who must anticipate where the shot might go. These considerations have been formulated at a qualitative level and have rarely been quantitatively tested in observational samples of Paralympic sports (53); the present data do not allow them to be tested either, and they should be regarded as hypotheses for future research.

In the analysis conducted, five players had AI = 100%, indicating the exclusive use of a single foot, and only two players used both feet regularly, with AI = 54.3% and 40.0%, respectively. This degree of specialisation suggests that, in F5, the executing foot is not merely a momentary technical choice but a stable individual pattern that is repeated across matches. Whether such unilateral patterns translate into asymmetric mechanical loading and a higher risk of overuse injury cannot be determined from the present data, as no injury, training-load, strength or biomechanical measures were collected. In sighted professional soccer, Nicholson et al. (54) reported that kicking-limb preference is associated with interlimb differences in the hamstring-to-quadriceps (H:Q) ratio, with potential implications for players’ health and performance. If comparable associations were confirmed in F5, intervention plans with individualised approaches (55, 56) tailored to the needs of the specific context would be warranted, especially in athletes who require unilateral force application (57); at present, this should be regarded as a hypothesis requiring prospective testing.

### 4.1 Limitations and future directions

This study has certain limitations that must be considered. First, the sample was small: 141 actions, eight players, six matches and only 14 goals (12 in the multivariable models), which results in low statistical power, wide CIs and ≈1.7 events per parameter in the full model. Although penalised estimation and a reduced model were used to mitigate small-sample bias, all adjusted estimates should be regarded as exploratory. Second, actions were nested within players and matches. The random-intercept model with player as the clustering unit converged when estimated via Gauss–Hermite quadrature, but with only eight players, five of whom used a single foot, the executing foot is strongly confounded with player identity; the player-level variance and the foot effect are therefore weakly identified, and the resulting interval was very wide. Crossed player and match effects could not be estimated reliably. Third, limb dominance was not assessed independently (e.g., via questionnaires or standardised kicking tests); foot preference was inferred from competitive behaviour, which is itself the object of study. Fourth, the data came from a third-party public database: the actions and court coordinates were not coded by the authors, and no information on intra- or inter-observer reliability or on the measurement error of the spatial digitisation is available. Because distance, angle and zones were derived from these coordinates, measurement error may have attenuated spatial associations. In addition, the left–right orientation of the digitisation template could not be verified, so lateral position was analysed as central versus wide, and no start-of-play coordinates were available, which precluded the analysis of progression or ball trajectory. Fifth, anthropometric, physical, physiological and biomechanical variables, as well as defensive pressure and goalkeeper positioning, were not available. Finally, the analysis focuses solely on the offensive actions of a single national team in a single tournament, without knowledge of the lateral play patterns of opposing teams or the strategies employed by opposing goalkeepers, which limits the generalisability of the results.

Future studies should consider larger samples spanning several teams and tournaments, ideally with independent assessment of limb dominance and reliability-tested coding of actions and court coordinates, which would allow Bayesian or frequentist hierarchical models with adequate numbers of players and events. Likewise, future studies require the synchronization of game data with other individual biomechanical variables, notably strength asymmetry and unilateral functional tests, which could help distinguish functional asymmetry in tactical terms from anatomical asymmetry when analysing F5. Future studies could also analyse women's F5, which has only recently been incorporated into international competition. The integration of longitudinal data on foot use, training load and injury incidence is needed to test whether unilateral patterns are associated with overuse injuries in athletes with visual impairments. Finally, further research is needed that uses machine learning models to analyse determinants of performance in F5 (58) and that integrates physical fitness, technical-tactical, physiological, and biomechanical variables.

### 4.2 Practice implications

Profiling each player's foot use can inform technical–tactical scouting, both of the team's own attackers and of opposing goalkeepers, and the design of bilateral technical training in F5, where the availability of a small roster (typically between 8 and 10 players) poses significant challenges in such short competitions. Whether the early identification of players with extreme AI, along with the quantification of their unilateral competitive load, should form the basis of individualised injury-prevention protocols is a hypothesis that requires prospective validation before it can be recommended.

Specifically, it is suggested that training programmes incorporate the evaluation of bilateral technical proficiency, the monitoring of unilateral load, technical analysis, and motor development to help improve individual and team performance in F5.

## 5 Conclusions

The Argentine men's national F5 team at the 2022 IBSA Copa América showed marked team-level right-foot predominance (AI = 51.8%; R:L ratio = 3.1:1) and pronounced individual specialisation, with five of the eight players using a single foot (AI = 100%). Left-foot actions had a higher crude goal rate than right-foot actions (17.6% vs. 7.5%; OR = 2.65; 95% CI: 0.85–8.28; *p* = 0.102) and similar adjusted estimates, but the association was imprecise, not statistically significant in any specification and largely driven by one left-footed player, and it was weaker when foot use was expressed relative to each player's preferred foot. Individual lateral diversity was not associated with goal-scoring rates, and shot distance and angle did not differ between feet.

These exploratory findings do not demonstrate that the non-preferred limb confers an offensive advantage; rather, they identify a hypothesis, namely the selective use of the non-preferred foot in favourable situations, that should be tested in larger, multi-team samples with independent assessment of limb dominance. Individual foot-use profiles may help coaches plan bilateral technical training and scouting in athletes with visual impairments, whereas any implications for unilateral load and overuse-injury risk remain to be established.

## Data Availability

The original contributions presented in the study are included in the article. Further inquiries can be directed to the corresponding author.
