# Supplementary material for: Lateral foot-use asymmetry and offensive effectiveness in elite 5-a-side soccer for the blind: an exploratory notational analysis
Source: Front Sports Act Living. 2026 Oct 21;8:1887871. doi: 10.3389/fspor.2026.1887871 (PMC13731249; doi:10.3389/fspor.2026.1887871)
Supplement: Supplementary file 1 [file Supplementaryfile1.docx]

Supplementary Figure S1.

Coordinate system and court zones. (a) Court coordinates in the source database: X is the lateral position across the court width (0–20 m; goal centre at X = 10 m), and Y is the longitudinal position, which for the Argentine team’s attacks equals the distance from the attacked goal line (Y = 0 m); d, distance from the shot location to the goal centre; θ, absolute shot angle relative to the goal axis. (b) Depth zones (0–6, 6–10 and 10–20 m) and lateral lanes (0–7, 7–13 and 13–20 m) of the attacking half. Supplementary Code S1. Python code that reproduces the data processing, analyses, tables and figures. Supplementary Data S1. Processed dataset of the 141 analysed actions.

**FIGURE S1.** Coordinate system and court zones.
